# Optimizing Nutritional and Functional Properties of Gluten‐Free Grass Pea and Quinoa Instant Flours: Dual Modification by Sprouting and Extrusion

**DOI:** 10.1002/fsn3.70697

**Published:** 2025-08-18

**Authors:** Mahnoosh Afsharfar, Zahra BeigMohammadi, Elnaz Milani, Seid Mahdi Jafari

**Affiliations:** ^1^ Department of Food Science and Technology NT.C., Islamic Azad University Tehran Iran; ^2^ Department of Food Processing Iranian Academic Center for Education Culture and Research (ACECR) Mashhad Khorasan Razavi Province Iran; ^3^ Department of Food Materials and Process Design Engineering Gorgan University of Agricultural Sciences and Natural Resources Gorgan Iran; ^4^ Halal Research Center of IRI Iran Food and Drug Administration, Ministry of Health and Medical Education Tehran Iran

**Keywords:** extrusion, germination, gluten‐free flour, grass pea, quinoa

## Abstract

The growing demand for gluten‐free products (GFP) among individuals with celiac disease and those adhering to gluten‐free diets has prompted research into alternative flour sources offering enhanced nutritional profiles. This study investigated how sprouting and extrusion affect the quality attributes of GFP‐based instant flour. The effects of feed moisture content (14%, 17%, and 20%) and whole sprouted grass pea (SGP): sprouted quinoa (SQ) blends (25:75, 50:50, and 75:25 w/w) on the macro and microstructure, functional properties, and nutritional properties of extrudates were analyzed using a central composite design. Results revealed that optimal conditions were 18.94% feed moisture content and a 75:25 SQ:SGP ratio. Under these conditions, significant increases were observed in porosity, water absorption index, and antioxidant properties, along with reduced hardness (*p* ≤ 0.05). The extruded flour exhibited improved particle size, protein, total dietary fiber, soluble fiber, antioxidant activity, and functional properties compared to the untreated flour. Notable improvements in flowability (Carr index and Hausner ratio), dispersibility, a* and b* attributes were observed in the extruded flour compared to the untreated flour.

## Graphical abstract

This study explores the impact of sprouting and extrusion on the quality of gluten‐free precooked flour made from grass pea and quinoa blends. Optimal conditions, including 18.94% feed moisture content and a 75:25 sprouted quinoa to grass pea ratio, significantly enhanced the nutritional and functional properties, such as increased porosity, water absorption, and antioxidant activity, while reducing hardness. The results highlight the potential of these dual modifications to improve the quality of gluten‐free products.

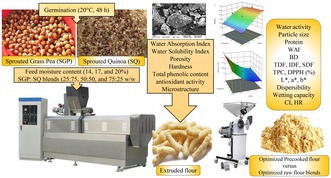

## 1 Introduction

The increasing prevalence of celiac disease and the growing adoption of gluten‐free diets have driven demand for gluten‐free products (GFPs). This increasing demand has motivated research into alternative flour sources with enhanced nutritional profiles (Comettant‐Rabanal et al. 2021). Various gluten‐free flours are utilized to create GFPs. Flours derived from various gluten‐free sources have become essential ingredients in a range of food applications, including weaning foods, breakfast cereals, porridge, desserts, and soups (Pismag et al. 2024). The instant flour industry is experiencing rapid growth, driven by advancements in processing technologies aimed at improving production efficiency (Akande et al. 2017).

Recent research has focused on utilizing legume flours in instant products due to their desirable nutritional and functional properties (Pismag et al. 2024). Legume flours are valuable sources of essential amino acids, minerals, and B‐complex vitamins. Legumes are recognized for their low‐fat content, absence of cholesterol, and beneficial properties, including water‐binding capacity, solubility, nutritional benefits, and antioxidant potential (AP) (Comettant‐Rabanal et al. 2021). Grass pea ( *Lathyrus sativus* ), an annual legume cultivated as a pulse crop, is valued for its rich nutritional profile. This profile includes high protein content 26%–34%, essential amino acids, polyunsaturated fatty acids, folic acid, and a significant total phenolic content (TPC) (Feyzi et al. 2017).

Grass pea ( *Lathyrus sativus* ) is a low‐cost, pharmacologically rich, and nutritionally dense crop with considerable potential in functional food formulations, aligning with global trends toward sustainable and health‐conscious nutrition. Emerging evidence highlights its bioactive potential, including a high protein content (20%–30%), richness in essential amino acids such as lysine and arginine, a low glycemic index, and antioxidant, anti‐inflammatory, and antimicrobial activities. Although β‐N‐oxalyl‐L‐α,β‐diaminopropionic acid (β‐ODAP) toxicity remains a challenge, modern processing techniques such as sprouting and extrusion effectively mitigate associated risks while enhancing health benefits. Consequently, grass pea is gaining increased attention for its functional benefits in gluten‐free products, plant‐based proteins, and therapeutic foods (Solovieva et al. 2025).

Quinoa ( *Chenopodium quinoa* wild), recognized as a “superfood,” complements legumes nutritionally, containing 14%–17% protein with a balanced essential amino acid profile, including methionine, cysteine, and lysine, which are often limiting in legumes. Furthermore, quinoa is a source of tocopherols, carotenoids (exhibiting antioxidant and potential anti‐cancer properties), dietary fiber, B vitamins (B_1_, B_2_, and B_6_), vitamin C, vitamin E, and minerals such as calcium, phosphorus, iron, and zinc. To enhance the functional, nutritional, and sensory attributes of these grains in GFPs, simple and cost‐effective processing methods, such as sprouting, roasting, autoclaving, fermentation, and extrusion cooking, have been employed (Albarracín et al. 2019; Kesselly et al. 2023).

Extrusion, a high‐temperature short‐time (HTST) processing technique, is effective for modifying raw material components, such as starch, protein, and fiber, particularly in instant flour (Milani et al. 2024). Its large‐scale industrial applicability and eco‐friendly nature, resulting from minimal water usage and waste generation, further contribute to its appeal. Sprouting, often combined with extrusion, has been shown to enhance the nutritional and sensory attributes of extrudates (Albarracín et al. 2019). Krapf et al. (2019) reported improvements in both nutritional quality and sensory characteristics of extrudates based on sprouted wheat flour, attributed to the formation of short‐chain sugars from starch breakdown during germination. Paucar‐Menacho et al. (2022) demonstrated that incorporating sprouted quinoa and cañihua flours increased phytic acid and antioxidant activity in corn grit‐based extrudates. Ajala et al. (2025) found that extrusion significantly improved the nutritional composition, enhancing protein digestibility and mineral bioavailability of snacks made from pearl millet and Bambara groundnut flour, while reducing anti‐nutritional factors such as trypsin inhibitors and phytates. Furthermore, increasing the proportion of Bambara groundnut flour elevated levels of protein, dietary fiber, and essential minerals (calcium, potassium, and iron) and simultaneously decreased carbohydrate and anti‐nutrient contents. These findings underscore that extrusion is not only versatile for value‐addition to various cereals and legumes but also a potent means of producing nutrient‐dense snacks to help combat malnutrition.

Limited information exists regarding the effects of combined sprouting and extrusion on the nutritional and functional characteristics of whole‐grain foods. Response Surface Methodology (RSM), a statistical approach widely used to optimize formulation and processing conditions by modeling variable interactions, was employed in this study (Toliaty et al. 2022). This study aimed to evaluate the effects of feed moisture content (FMC) and the ratio of quinoa and grass pea on the nutritional, physicochemical, and functional quality parameters of gluten‐free instant flour using RSM.

## 2 Materials and Methods

### 2.1 Materials

Whole grass pea seeds ( *Lathyrus sativus* ‘Bujan’) and dehulled quinoa ( *Chenopodium quinoa* ‘Sajama’) were obtained from the Agricultural Research, Education, and Extension Organization in Mashhad, Iran. The materials were analyzed for protein, fat, ash, total dietary fiber (TDF), soluble dietary fiber (SDF), insoluble dietary fiber (IDF), and moisture content (MC) according to AOAC methods (2000). Total carbohydrate content was calculated by subtracting the sum of protein, fat, ash, and MC from 100%. Water activity (*a*_*w*_) was measured at 25°C using a Rotronic AG Hygroscopic DT 2 with an accuracy of 0.001 *a*_*w*_ units (Afshani et al. 2019). All results are presented as means ± standard deviations (SD) of three independent analyses.

### 2.2 Method

#### 2.2.1 Preparation of Germinated Flour

The seeds were soaked in distilled water for 24 h, then incubated at 20°C for 48 h in an incubator (MAC, Model MWS‐231, New Delhi, India) to promote germination. Subsequently, the sprouted seeds were dried in a hot air oven (Everflow Scientific Instruments, Chennai, India) at 50°C for 12 h (Yarabbi et al. 2023). Dried samples were then milled using a Pilotsmith (India) Pvt. Ltd. pulverizer (Model No 412‐2, Kerala, India) to obtain a consistent particle size of ≤ 250 μm.

#### 2.2.2 Preparation of Instant Flour

Extrusion was performed using a pilot‐scale, co‐rotating, twin‐screw extruder (model DS56, Jinan Saxin, China) with a barrel length of 80 cm, a screw diameter of 16 mm, a maximum screw speed of 320 rpm, and a die diameter of 4 mm. Preliminary tests established the following extrusion parameters: barrel temperature of 140°C, screw speed of 160 rpm, and feed rate of 40 kg/h. The extrudates were then dried in an air oven at 55°C for 2 h, cooled, and packaged in laminated pouches for further analysis. A portion of the extrudates was subsequently milled under the same conditions as the raw materials for further analysis (Okhravi et al. 2024).

#### 2.2.3 Functional Properties

To determine the Water Absorption Index (WAI) and Water Solubility Index (WSI) of the extrudates, the method described by Lotfi Shirazi et al. (2020) was followed. Briefly, 2.5 g of sample was dispersed in 25 mL of distilled water and stirred magnetically for 30 min. The resulting suspension was then centrifuged at 3000 rpm for 15 min using a centrifuge (UniCen, Herolab, Germany). The supernatant was subsequently dried in an oven (FD 115, BINDER, Germany) at 100°C for 8 h. WAI and WSI were calculated according to Equations (1) and (2).

(1) $$\mathrm{WSI}\;\left(\%\right)=\frac{\text{Weight of dissolved solids in supernatant}}{\text{Weight of sample}}\times 100$$

(2) $$\mathrm{WAI}\;\left(g/g\right)=\frac{\text{Weight of hydrated sediment}\;}{\text{Weight of sample}}$$

#### 2.2.4 Porosity

The gas pore and microstructure of the extrudates were assessed using an image processing system. Images were acquired with a digital camera (Canon EOS 1000D) at a resolution of 5184 × 3456 pixels and 150 ppi pixel depth. The extrudates were longitudinally cut with a cutter. The total number of measured objects per sample ranged from 700 to 1500, and analysis was quantified using ImageJ software (National Institutes of Health, Bethesda, MD, USA), following the method described by Saldanha do Carmo et al. (2019).

#### 2.2.5 Hardness

The hardness of the extrudates, expressed in Newtons (N), was measured using a texture analyzer equipped with a 50 kg load cell (TA‐XT2i Plus, Stable Micro Systems Ltd., UK). Three randomly selected samples from each treatment were analyzed by measuring the maximum force required to penetrate the sample with a 10 mm diameter cylindrical probe at a constant speed of 1 mm/s (Lotfi Shirazi et al. 2020).

#### 2.2.6 Total Phenolic Content

Total phenolic content (TPC) was determined using the Folin–Ciocalteu spectrophotometric method described by Sarawong et al. (2014), with minor modifications. Briefly, 0.2 mL of the sample extract or gallic acid standard solution was mixed with 1.5 mL of a tenfold‐diluted Folin–Ciocalteu reagent. After 5 min, 1.5 mL of sodium carbonate solution was added, and the mixture was incubated in the dark at room temperature for 90 min. Absorbance was measured at 725 nm using a UV‐1800 spectrophotometer. A mixture of acetone and water (80:20, v/v) served as the blank, and gallic acid was used for calibration. Results were expressed as mg gallic acid equivalents (GAE) per 100 g of sample. All measurements were performed in triplicate.

#### 2.2.7 Antioxidant Potential Measured by DPPH

Antioxidant potential (AP) was assessed using the 2,2‐diphenyl‐1‐picrylhydrazyl (DPPH) radical scavenging assay, following the method of Hashemi et al. (2024) with minor modifications. Briefly, 1 g of the dried sample was ground, sieved, and extracted with 20 mL of 80% (v/v) methanol. The mixture was vortexed to ensure homogenization and incubated in a shaking water bath at 37°C for 2 h. After incubation, the extract was centrifuged at 4000 rpm for 10 min, and the resulting supernatant was collected and stored at 4°C for subsequent analysis. DPPH radical scavenging activity was calculated based on absorbance measurements and expressed as a percentage of inhibition using Equation (3). All assays were conducted in triplicate, and results are reported as mean ± standard deviation (SD).

(3) $$\mathrm{AP}=\left[1-\left(\frac{\text{Absorbance Sample}}{\text{Absorbnance Control}}\right)\right]\times 100$$

#### 2.2.8 Color Properties

The color of the extrudate powders was measured using a HunterLab ColorFlex EZ spectrophotometer (HunterLab, Reston, VA, USA). Before measurement, extrudates were ground and sieved. The resulting powders were then placed in the instrument's sample cup to ensure even distribution, and measurements of lightness (L*), redness (a*), and yellowness (b*) were recorded.

#### 2.2.9 Microstructure of the Extrudates

The microstructure of the extrudates was examined using a scanning electron microscope (SEM) (LEO 1450VP, ZEISS, Germany). Samples were mounted on stubs, sputter‐coated with a gold–palladium layer for 3 min at 5 mA, and then imaged at various magnifications using an accelerating voltage of 20 kV (Hashemi et al. 2024).

#### 2.2.10 Wetting Capacity

Wetting capacity was determined by measuring the time required for 1 g of powder to fully submerge when gently placed onto 100 mL of distilled water at 20°C (Barbosa 2021).

#### 2.2.11 Dispersibility

Dispersibility was assessed using a modified procedure based on Barbosa (2021). Briefly, 15 g of sample was added to a 100 mL graduated cylinder filled with distilled water, stirred for 90 s, and allowed to settle for 15 min. Dispersibility was calculated as the difference between the initial volume (100 mL) and the volume of settled particles, expressed as a percentage.

#### 2.2.12 Particle Size

The particle size of the extruded flours was determined using dynamic light scattering (Horiba SZ‐100, Horiba Ltd., Kyoto, Japan) at room temperature. The average particle size was reported as the volumetric diameter (Hashemi et al. 2024).

#### 2.2.13 Bulk Density, Hausner Ratio (HR) and Carr Index (CI)

The flow behavior of powder is evaluated using the CI and HR. Both HR and CI are numerical values that depend on the bulk density and tapped density of the powder (Nabavi et al. 2024). These parameters are calculated as follows:

(4) $${\text{Hausner}}^{\prime }s\;\text{ratio}\;\left(\mathrm{HR}\right)=\frac{\mathrm{\rho T}}{\mathrm{\rho B}}$$

(5) $${\text{Carr}}^{\prime }s\;\text{Index}\;\left(\mathrm{CI}\right)\%=\frac{\mathrm{\rho T}-{\mathrm{\rho B}}^{\prime }}{\mathrm{\rho T}}\times 100$$

The tapping density is determined by tapping a graduated cylinder 100 times on a bench until the powder settles and no further settling is observed. The tapping process involves an average of two taps per second. The density is calculated using the formula, where *V*_*t*_ represents the volume occupied after tapping (m^3^):

(6) $$\mathrm{\rho T}=\frac{W_{s}}{V_{t}}$$

Particle density was measured using a helium multipycnometer (Quantachrome MVP 4AC232). The procedure involves flowing helium under pressure from the reference cell into a cell containing the sample material. The particle density is determined by calculating the ratio of the mass of the sample (*W*_*s*_ in kg) to the average particle volume (*V*_*p*_ in m^3^):

(7) $$\mathrm{\rho p}=\frac{W_{s}}{V_{p}}$$

### 2.3 Experimental Design

A central composite design (CCD) was employed to investigate the interaction between the independent variables: sprouted quinoa (SQ): sprouted grass pea (SGP) ratio (25:75 to 75:25) and FMC (14%–20%). Table 1 presents the coded and actual values of these variables. Data were analyzed using Design‐Expert software (version 7.0.1, Stat‐Ease Inc., Minneapolis, MN, USA). The most suitable model for evaluating the influence of the variables on the responses was selected. Analysis of variance (ANOVA) was performed to assess model adequacy, using the coefficient of determination (*R* ^2^), adjusted *R* ^2^, *F*‐value, and lack‐of‐fit test. The fitted model for each response is represented by Equation (6):

(8) $$Y_{i}=b_{0}+\sum b_{i}x_{i}+\sum b_{\mathrm{ii}}{x_{\mathrm{ii}}}^{2}+\sum b_{\mathrm{ij}}x_{i}x_{j}$$

Where *Y*_*i*_ is the response variable, *x*_*i*_ and *x*_*j*_ are the coded values of the independent factors, *b*_0_ is the intercept, and *b*_*i*_, *b*_*ii*_, and *b*_*ij*_ are the linear, quadratic, and interaction coefficients, respectively.

**TABLE 1:** Coded and actual values of independent variables.

Independent variable	Symbol	Code level		
		−1	0	+1
SQ: SGP	X_1_	25:75	50:50	75:25
Feed moisture content (%)	X_2_	14	17	20

Regression analysis and ANOVA were performed to identify the most suitable model and assess the statistical significance of the responses. Model adequacy was evaluated using the coefficient of determination (*R* ^2^) and the coefficient of variation (CV). Non‐significant lack‐of‐fit (*p* > 0.05) for all responses (Table 2) indicated adequate model fit for prediction.

**TABLE 2:** The coefficients of the variables in the proposed model for the response variables.

Parameter	WAI	WSI	Porosity	Hardness (N)	TPC (mg/100 g)	AP (%)
A‐SQ: SGP	0.1940	0.0031	16.07	14.17	0.0016	0.2601
B‐moisture	0.0080	0.5361	8.26	1.35	0.0017	0.3056
AB	0.0025	0.0110	6.25	1.56	0.0016	0.3025
A^2^	0.1798	0.1130	14.09	15.01	0.0020	0.3202
B^2^	0.0084	0.0664	8.54	0.1507	0.0163	0.2663
*Model*						
*F* value	0.0001***	0.0001***	0.0009***	0.0001***	0.0001***	0.0001***
*R* ^2^	0.9721	0.980	0.909	0.953	0.912	0.976
Adjust *R* ^2^	0.9230	0.990	0.825	0.907	0.896	0.911
Lack of fit	0.8008	0.8066	0.2006	0.5365	0.3100	0.6115

*Note:* ***significant at *p* < 0.001.

The objective of CCD is to identify best combination of independent process variables that either maximizes or minimizes the target response variable. This optimization is typically achieved through numerical methods, providing a robust statistical framework for optimizing process parameters.

The functional and nutritional characteristics of the optimized instant flour influenced by sprouting and extrusion processing were evaluated in comparison to the optimally raw material using a *t*‐test analysis with a 95% confidence level (Table 4). The values display means ± SD from three replicates, with different superscripts indicating significant differences (*p* < 0.05).

## 3 Results and Discussion

### 3.1 Comparative Evaluation of Chemical Composition

Table 3 details the significant changes in the chemical composition of quinoa and grass pea flours following sprouting. Both sprouted quinoa (SQ) and sprouted grass pea (SGP) exhibited a significant increase in moisture content, reflecting water absorption during germination. Protein content also significantly increased in both SQ (from 12.07% to 12.86%) and SGP (from 26.73% to 27.80%), attributable to the activation of proteolytic enzymes breaking down storage proteins into more bioavailable forms (Lakshmipathy et al. 2024). Contrasting trends were observed for carbohydrates: SQ showed a significant increase (from 71.12% to 72.99%), while SGP experienced a significant decrease (from 59.91% to 56.32%), suggesting different metabolic utilization patterns during germination. Concurrently, fat content significantly decreased in both flours (SQ: 5.43% to 5.06%; SGP: 3.02% to 2.94%), indicative of lipid utilization for energy during respiration (Albarracín et al. 2019; Paucar‐Menacho et al. 2022). Furthermore, crude fiber and ash content significantly increased in both sprouted samples, implying enhanced fiber fractions (Lakshmipathy et al. 2024) and improved mineral bioavailability, respectively. Notably, while TPC showed no statistically significant change (Table 3), DPPH radical scavenging activity significantly increased for both SQ (from 32.15% to 37.16%) and SGP (from 25.04% to 27.86%). This enhancement in AP, despite stable TPC levels, suggests the release or formation of other antioxidant compounds, or increased accessibility of existing ones, during the sprouting process (Yarabbi et al. 2023; Sofi Sajad et al. 2020).

**TABLE 3:** The physicochemical and nutritional analyses of the Gluten‐Free Whole Grain Flours (Dry Weight Basis).

Parameters	Raw quinoa (RQ)	Sprouted quinoa (SQ)	Raw grass pea (RGP)	Sprouted grass pea (SGP)
Moisture content %	6.89 ± 0.14^b^	8.02 ± 0.21^a^	8.58 ± 0.30^b^	9.93 ± 0.14^a^
Carbohydrates %	71.12 ± 1.12^b^	72.99 ± 1.03^a^	59.91 ± 1.19^a^	56.32 ± 1.17^b^
Protein %	12.07 ± 0.11^b^	12.86 ± 0.17^a^	26.73 ± 0.20^b^	27.80 ± 0.18^a^
Fat %	5.43 ± 0.17^a^	5.06 ± 0.19^b^	3.02 ± 0.01^a^	2.94 ± 0.01^b^
Crude fiber %	3.97 ± 0.19^b^	4.10 ± 0.57^a^	2.44 ± 0.29^b^	2.83 ± 0.24^a^
Ash %	3.71 ± 0.15^b^	4.03 ± 0.16^a^	2.96 ± 0.09^b^	3.08 ± 0.03^a^
TPC (mg GAE/100 g)	14.09 ± 0.86^a^	14.93 ± 1.03^a^	13.56 ± 0.36^a^	14.22 ± 0.49^a^
DPPH (%)	32.15 ± 1.08^b^	37.16 ± 0.73^a^	25.04 ± 0.90^b^	27.86 ± 1.02^a^

*Note:* Values are means ± standard deviations (*n* = 3); Means within a row with different superscript letters (a, b) are significantly different (*p* < 0.05).

### 3.2 Water Absorption Index

**FIGURE 1:**
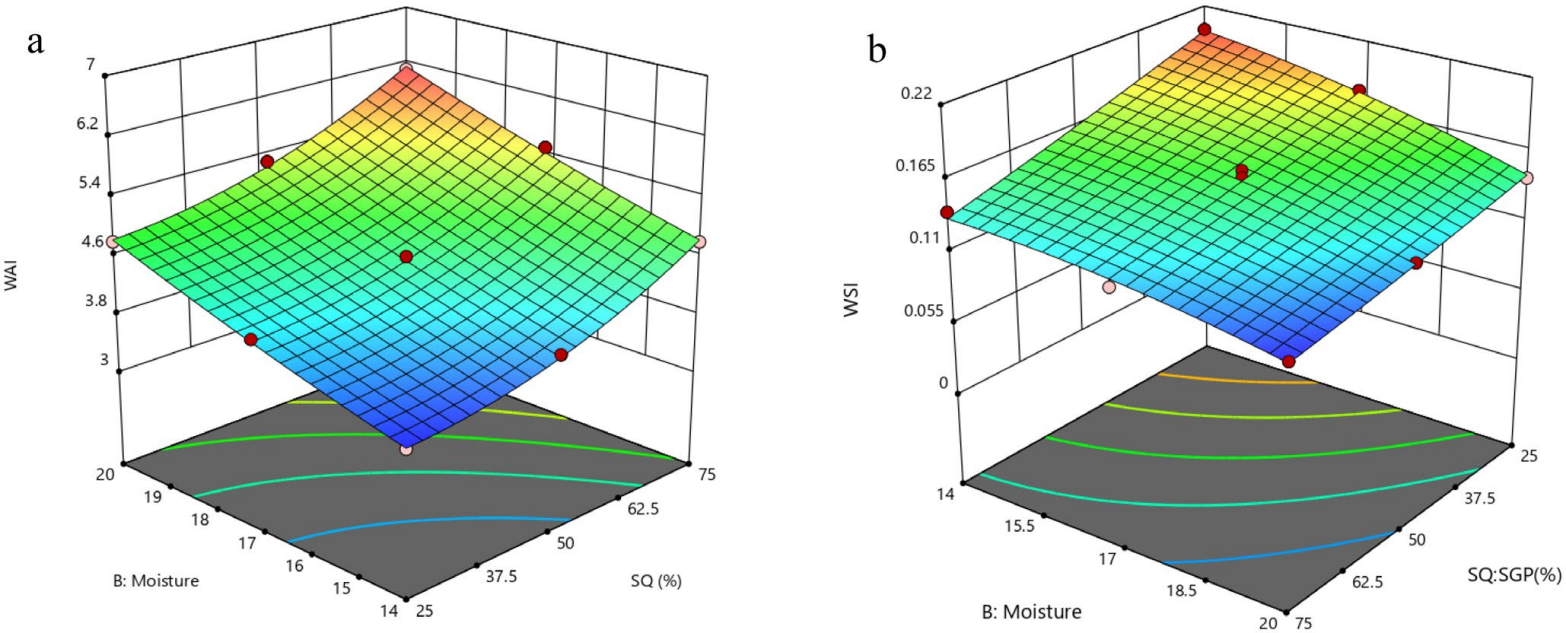
Effect of SQ: SGP ratio and FMC on (a) WAI and (b) WSI of instant flours.

WAI reflects the capacity of the extruded product to form a gel with water, representing the moisture retention capability of pre‐gelatinized starches (Chou and Hsu 2021). In this study, the WAI of the extruded flours ranged from 3.6 to 6.1 g/g. Figure 1a illustrates the effects of the SQ:SGP ratio and FMC on WAI. Increased feed moisture content (FMC), along with rising SQ levels during extrusion, leads to higher WAI values. The higher moisture content plasticizes the starch‐protein matrix, facilitating gelatinization, molecular realignment, disruption of crystalline structures, and the exposure of hydrophilic groups (Ciro Baruchs et al. 2024). This increased moisture content reduces melt viscosity, which enhances expansion and creates a more porous microstructure, ultimately improving water penetration (Boluk et al. 2023). Additionally, a higher starch content in SQ undergoes shear‐induced gelatinization during extrusion, which increases the number of hydrophilic sites. Furthermore, soluble fiber in SQ absorbs water and contributes to an increase in viscosity (Yarabbi et al. 2023; Muñoz‐Pabon et al. 2022). The maximum WAI was observed at 20% FMC and a 75% SQ level. This phenomenon can be explained by the role of water as a plasticizer, which limits starch degradation during extrusion (Boluk et al. 2023). The increased FMC may also enhance the bioavailability of hydrophilic groups, further contributing to higher WAI (Hashemi et al. 2024).

Throughout the extrusion process, gelatinization affects the molecular interactions within starch structures by disrupting both intermolecular and intramolecular bonds in the amorphous and crystalline regions, which exposes hydroxyl groups that can form hydrogen bonds with water (Boluk et al. 2023). WAI is affected by interactions with low‐molecular‐weight compounds generated during starch gelatinization and protein denaturation (Saldanha do Carmo et al. 2019). These interactions lead to new complexes with hydrophilic characteristics, increasing WAI. The rise in WAI and the increasing level of SQ in the instant flour formulation could also be attributed to the higher fiber content. Fiber content significantly increases WAI due to its ability to form gels and hold water. Similar observations were reported for instant grain amaranth‐based porridge flour (Akande et al. 2017) and maize‐mung bean‐based instant weaning food (Ali et al. 2016).

At higher FMC, the moisture lubricates the interior, reducing the extent of cooking. This reduces the solubility of starch at room temperature, which lowers the WAI at high FMC.

The equation formulated for estimating WAI is as follows:

(9) $$\mathrm{WAI}=2.761+0.0211\;A+0.01398\;B+0.003\;\mathrm{AB}++0.004\;A^{2}++0.0061\;B^{2}$$

### 3.3 Water Solubility Index

WAI and WSI are indirect indicators of starch conversion in raw materials. The WSI refers to the ratio of water‐soluble components in the extruded material, expressed as a percentage of the dry weight. This measurement is linked to the degree of starch dextrinization level during the extrusion process. Furthermore, WSI assesses the solubilization capability of instant flours or food powders in solvents. The characteristics of this property vary based on the molecular weight of the soluble fractions and their ability to form hydrogen bonds with water (Solovieva et al. 2025). Table 2 shows that WSI varied between 18% and 35.5%. The *F*‐value for the WSI was 0.0001, while the lack‐of‐fit was recorded at 0.8066. The *R* ^2^ and adjusted *R* ^2^ were 0.98 and 0.99, respectively, indicating a high coefficient of determination. Figure 1b shows that an increase in WSI was observed in samples with lower FMC, likely due to the rise in extrusion temperature, which encourages the cross‐linking of shorter units (Krapf et al. 2019; Sofi Sajad et al. 2020). This temperature enhances starch gelatinization, facilitates the leaching of amylose, and promotes dextrinization by breaking down starch into smaller molecules. In contrast, higher MC leads to noticeable solubility losses (Comettant‐Rabanal et al. 2021). An increase in MC negatively impacts shear forces, reducing the mean residence time of the extruded material in the extruder, thus limiting the thermo‐mechanical modification of the available starch and other macromolecules (Ali et al. 2016).

The Water Solubility Index (WSI) is directly influenced by the extent of starch polymer degradation, whereby macromolecular cleavage releases low‐molecular‐weight soluble polysaccharides into the aqueous phase. The WSI decreased with increasing SQ levels. As shown in Table 2, this can be attributed to the higher content of starch in SQ versus SGP, leading to the improvement of gelatinization and a decrease in the soluble fraction of starch (Akande et al. 2017). During the extrusion process, hydrothermal and mechanical factors lead to denaturation and aggregation of proteins, resulting in lower solubility (Sofi Sajad et al. 2020; Ai et al. 2016). Solubility was reduced by increasing MC in the mixture from 14% to 20% and the SQ content at the same time. When exposed to high MC, proteins unfold from their quaternary structures and integrate into the molten mass. These modified proteins then polymerize, crosslink, and reorient themselves, forming more extended and fibrous structures. This phenomenon is associated with changes in the properties of starch and proteins as well as a decrease in water solubility (Ai et al. 2016; Ali et al. 2016).

The equation formulated for estimating WSI is as follows:

(10) $$\mathrm{WSI}=0.2063-0.0027\;A-0.01137\;B+0.00009\;\mathrm{AB}+2.0551\;A^{2}-0.0006\;B^{2}$$

### 3.4 Porosity

Porosity is a crucial quality parameter for factors such as packing, transportation, and process efficiency (Cheng et al. 2020). Also, the mechanical properties of extruded flours are mainly driven by their bulk density and porosity, where the lower the bulk density and higher the porosity, the less the cost of packaging. In this investigation, the porosity of the samples was found to vary from 29.4% to 58.3%, with the highest porosity recorded at an SQ: SGP ratio of 25:75 and FMC of 20%. The *F*‐value for the porosity was 0.0009, while the lackoffit was recorded at 0.2006. The *R* ^2^ and adjusted *R* ^2^ values were 0.90 and 0.82, respectively (Table 2), indicating a high coefficient of determination. Throughout the extrusion process, the heated dough experiences a rapid change in pressure, leading to puffing and a decrease in the bulk density of the extruded flour. Powders with low bulk density exhibit high porosity, which increases their interaction with water molecules and enhances the wettability of instant flour (Ali et al. 2016; Saldanha do Carmo et al. 2019; Pismag et al. 2024).

**FIGURE 2:**
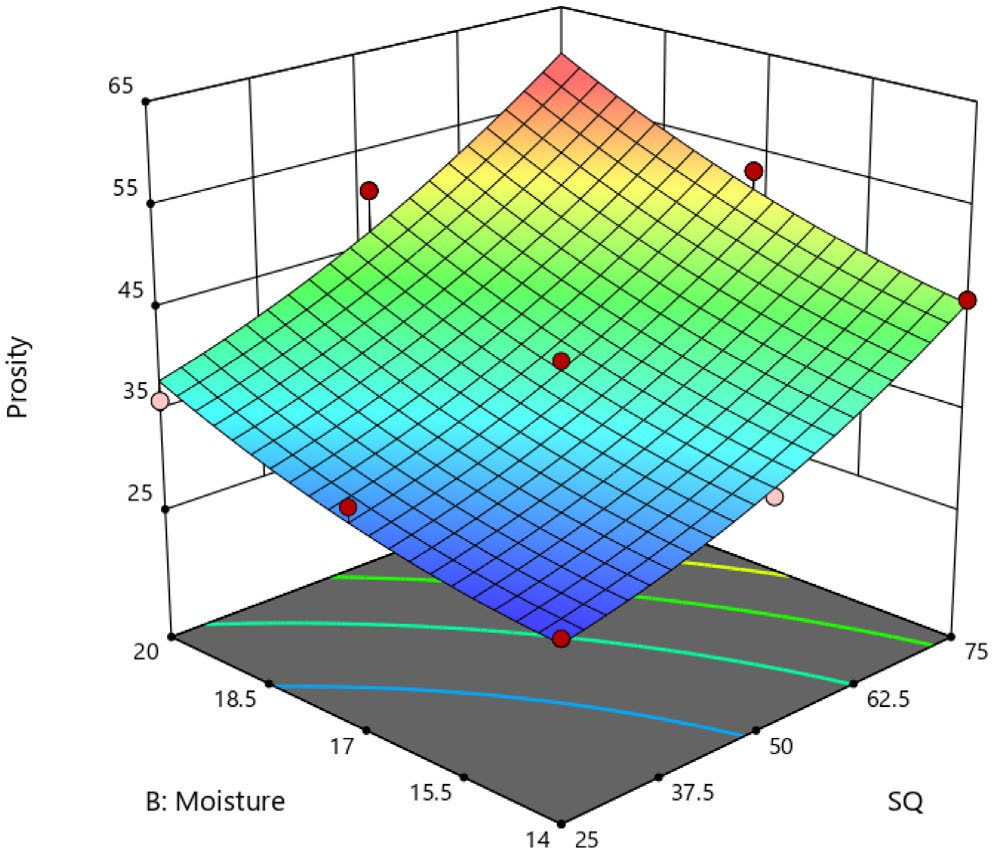
Effect of SQ: SGP ratio and FMC on the porosity of instant flours.

Figure 2 shows how the SQ:SGP ratio and FMC impact the porosity of the extrudates. Increasing the proportion of SGP in the blend from 25% to 75% led to a decrease in porosity. Higher protein levels in SGP resulted in interactions between protein and water, as well as between protein and starch, which can adversely affect both starch gelatinization and the porosity of the extruded products (Kesselly et al. 2023). Additionally, an increased protein content in grass pea disrupts gas bubble walls through water absorption, leading to a reduction in porosity (Lakshmipathy et al. 2024).

The porosity significantly increased as FMC rose from 14% to 20% (Figure 2). According to existing literature, FMC is a crucial factor influencing the porosity and density of the extrudate, likely due to the plasticization of the melt, which reduces the dough's elasticity. Additionally, higher MC can enhance starch gelatinization by increasing friction between the extruder and the dough and modifying the amylopectin networks in starch‐based materials, thus boosting the porosity of the extrudates (Ali et al. 2016). During the extrusion process, as the molten material leaves the die, the rapid transition from high internal pressure to atmospheric pressure leads to a rapid loss of internal moisture. This sudden loss facilitates bubble formation, which becomes trapped within the melted material, resulting in extrudates with high porosity (Ali et al. 2016; Chou and Hsu 2021).

The equations for the fitted models, after disregarding the influence of non‐significant factors for the uncoded form of the process variables, were as follows:

(11) $$\text{Porosity}=65.076+0.251\;A+4.572\;B+0.16\;\mathrm{AB}+0.003\;A^{2}+0.196\;B^{2}$$

### 3.5 Total Phenolic Contents and Antioxidant Potential Measured by DPPH

**FIGURE 3:**
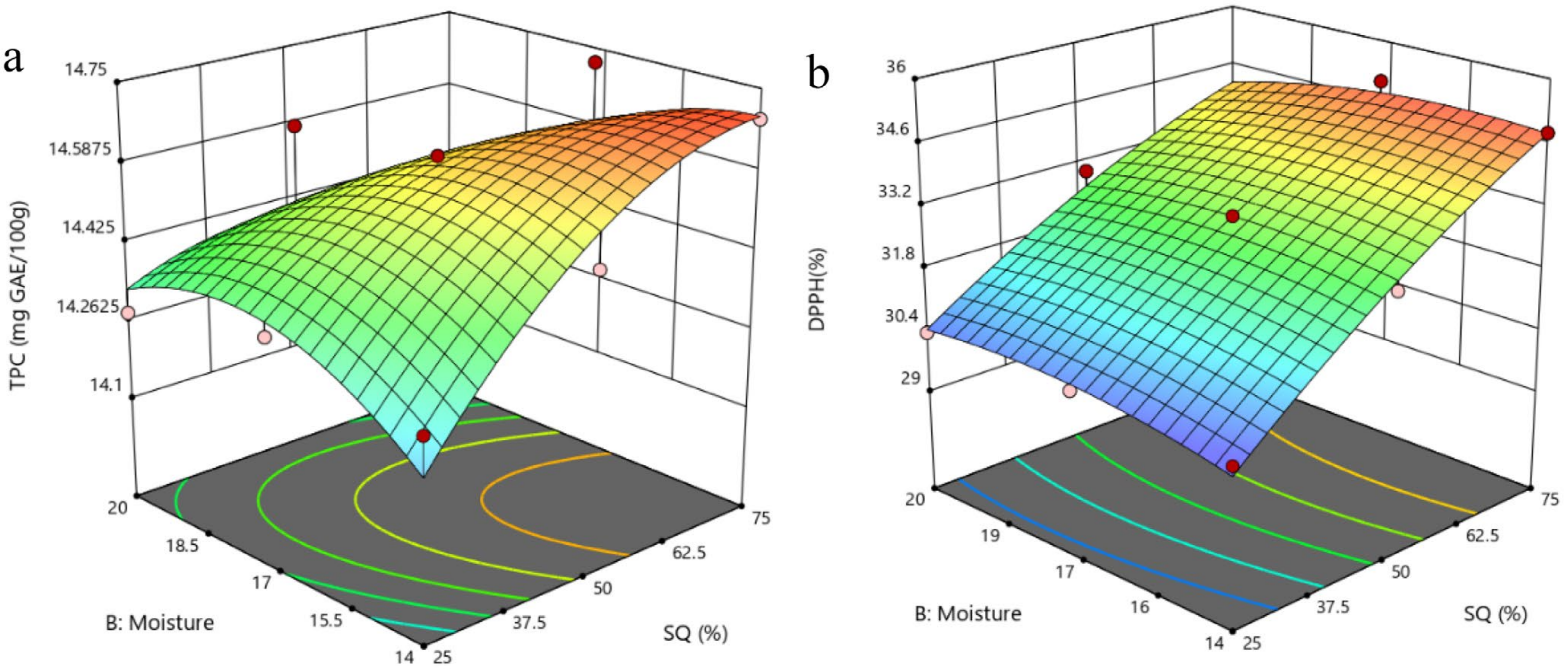
Effect of SQ: SGP ratio and FMC on (a) TPC and (b) AP of instant flours.

Figure 3a illustrates how incorporating SQ (%) and FMC influences TPC, while Figure 3b demonstrates the impact of current parameters on AP of instant flour. In this study, TPC and AP among the samples ranged from 14.28 to 14.72 (mg GAE/100 g) and 30.18 to 35.07 (%), respectively. ANOVA results from the regression analysis (Table 2) indicated that the quadratic terms related to SQ: SGP content, and FMC, significantly affected the TPC of the extrudates (*p* < 0.05). The high *R* ^2^ = 0.912 suggested that the model effectively captures their relationships (Equation (11)). Additionally, the influence of extrusion parameters on the antioxidant potential of the extruded flours was monitored using Equation (12). It was noted that the *R* ^2^ (0.896) for antioxidant potential of the product was satisfactory. The insignificance of the lack of fit suggested that the established second‐degree polynomial equation could effectively predict the relationship.

(12) $$\begin{array}{c} \mathrm{TPC}\;\left(\mathrm{mg}\;\mathrm{GAE}/100\;g\right)=11.509+0.0160\;A+0.2970\;B\\ \;-0.0002\;\mathrm{AB}-0.00004\;A^{2}–0.008\;B^{2} \end{array}$$

(13) $$\mathrm{AP}\;\left(\%\right)=14.516+0.202\;A+1.340\;B-0.003\;\mathrm{AB}-0.0005\;A^{2}–0.034\;B^{2}$$

The influence of process variables such as feed SQ content and MC on TPC (Figure 3) increased until it reached 75% SQ and 17% MC, with 100% TPC retention in the current sample; further increases in FMC led to a significant decline in TPC. A significant quantification of TPC has been observed when the conditions of extrusion effectively disrupt the cell walls of the matrix (Gulati et al. 2016). During the extrusion process, an increase in temperature, influenced by FMC < 17%, enhances the thermo‐mechanical movement of the molten material within the extruder, facilitating the release, extraction, and measurement of phenolic compounds including carotenoids, flavanols, and dietary fiber that were previously bound, thereby becoming part of the free phenolic fraction (Hashemi et al. 2024). It has also been noted that a rise in MC aids in preserving the released phenolic fractions, as lower MC during extrusion can lead to structural disruption or changes in phenolic compounds (Cheng et al. 2020).

It is crucial to note that some research shows a rise in the TPC of the extrudates, while others show a decrease, indicating that the outcome could vary based on the composition of the raw material and the extrusion conditions used (Cheng et al. 2020). Enhancing the incorporation of SQ led to a notable rise in TPC. Studies have shown that SQ and SGP are important sources of phytochemicals, particularly phenolic acids, anthocyanins, and carotenoids (Cheng et al. 2020; Corrales‐Bañuelos et al. 2016).

The AP of extruded products depends not only on the quantity of bioactive compounds but also on their specific composition. In this study, the DPPH (%) values varied between 30.18 and 35.06. Figure 3a illustrates how feed composition and MC influence AP as measured by DPPH, with the highest AP observed at a higher level of SQ (%) and FMC = 17 g/100 g. Conversely, the lowest AP was recorded with 25% SQ and FMC = 14 g/100 g. When FMC was increased to 20 g/100 g and SGP incorporation was raised to 75%, the ability to neutralize the DPPH radical diminished.

The AP diminishes when FMC falls below 14 g/100 g. It could be attributed to the increase in barrel temperature, which affected the molt dough behavior. During extrusion, the temperature of baking is a crucial factor in the stability of phytochemical compounds. It has been suggested that elevated temperatures during extrusion can harm phenolic compounds and reduce AP as well (Paucar‐Menacho et al. 2022). Occasionally, the concentration of bioactive compounds in extruded products may rise; for instance, the levels of ferulic acid in extruded cereal grains are reported to increase threefold, which impacts AP (Sarawong et al. 2014). The denaturation of grain proteins during the extrusion process encourages the interaction of tannins with proteins, leading to the formation of tannin‐protein complexes that preserve AP (Ai et al. 2016).

Hashemi et al. (2017) indicated that the enhancement of AP might result from the release of antioxidant phenolic compounds during the extrusion process, the inhibition of oxidation of phenolic compounds in the extrudate through enzymatic inactivation during processing, and the formation of Maillard reaction products. The availability of amino acids and reducing sugars in the raw materials, coupled with the application of high temperatures during processing, facilitates the creation of high molecular weight complexes, such as melanoidins, which have been linked to AP. Several researchers have noted that the presence of melanoidin precursors in extrudates led to an increase in AP after extrusion in quinoa and sesame by‐products, respectively (Gulati et al. 2016).

### 3.6 Hardness

**FIGURE 4:**
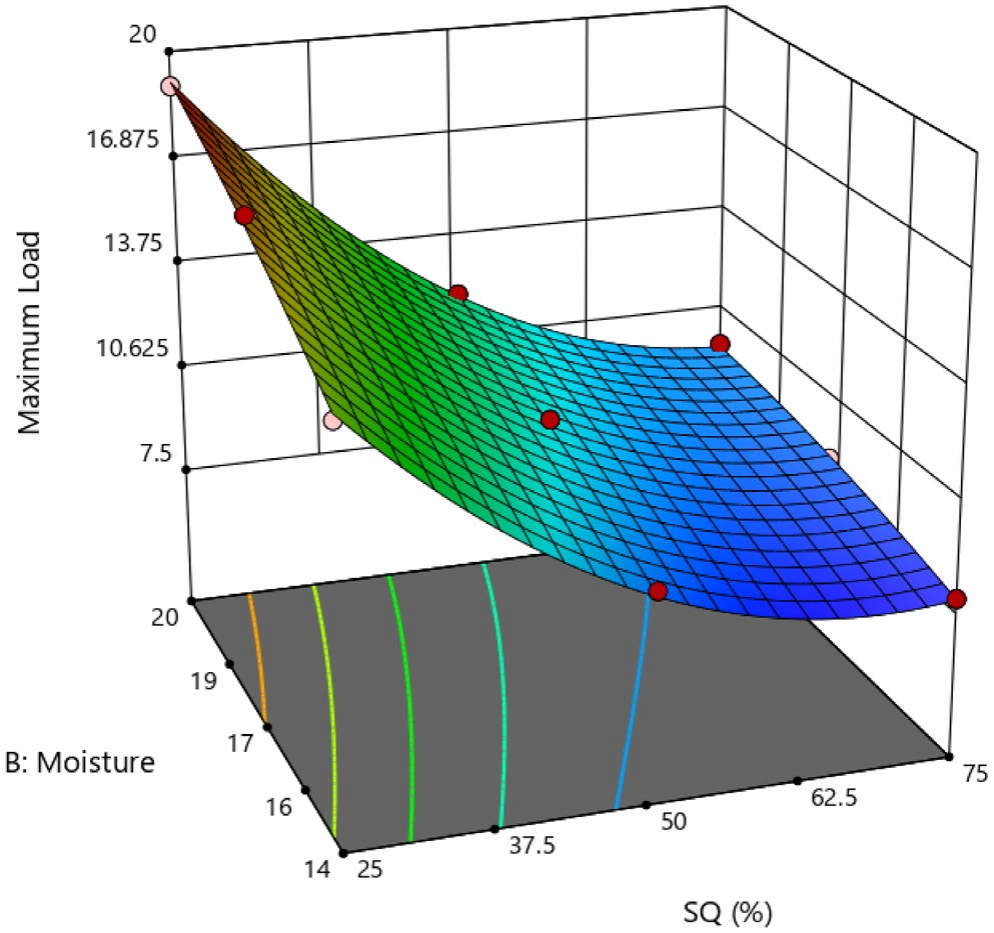
Effect of SQ: SGP ratio and FMC on the hardness of instant flours.

The way consumers perceive the hardness of samples is linked to the porosity and cellular structure of the extruded material (Cheng et al. 2020). The regression analysis regarding the hardness parameters of the extrudates with the incorporation of SQ: SGP and FMC is presented in Table 2. The hardness of the extrudates ranged from 7.9 N to 19 N. By improving FMC, the expanded mixtures' hardness also rose (Figure 4). A positive impact of MC on hardness indicates that higher FMC can adversely influence the expansion of extrudates, affecting their textural properties. The findings align with those of Ai et al. (2016) and Ali et al. (2016), who observed a rise in the hardness of bean‐based expanded flour and maize mung bean‐based instant weaning food as FMC increased, respectively. Moisture acts as a lubricant and reduces the shear force and process temperature, slowing the melting rate of the molten material and resulting in a low porosity and high hardness of extrudates (Lotfi Shirazi et al. 2020). Moreover, high MC limits the growth of bubbles and air cells in the product, causing compression of bubbles and reducing the gelatinization of starch, resulting in a denser and harder texture.

As illustrated in Figure 4, the hardness notably diminished with increasing levels of SQ. The presence of more polysaccharides and fiber in SQ correlated with enhanced crispness, porosity, and reduced hardness. Akande et al. (2017) reached similar findings on an instant grain amaranth‐based porridge flour. The increase in hardness attributed to the significant presence of SGP is linked to its protein content, which leads to a decrease in cell wall thickness and accelerates its breakdown. This observation aligns with research on cowpea flour affected by soaking and extrusion processes (Kesselly et al. 2023).

The model that has been fitted is presented below:

(14) $$\text{Hardness}\;\left(N\right)=+6.03–0.4001\;A+1.765\;B-0.008\;A\times B-4.07\;A^{2}+0.025\;B^{2}$$

### 3.7 Morphology and Microstructure

**FIGURE 5:**
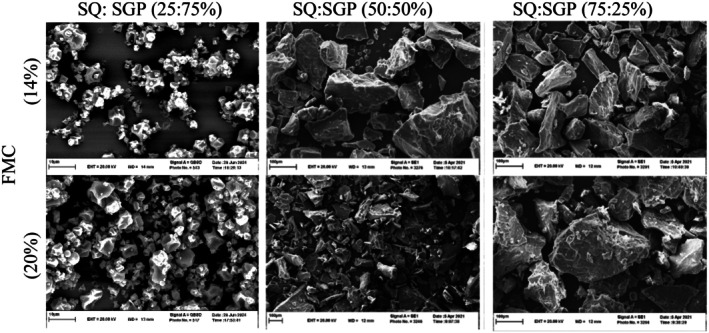
Scanning electron micrographs of extrudates produced with varying SQ: SGP ratios and FMC (14% and 20%) at 2000× magnification.

SEM images analyze the microstructure, focusing on the size, quantity, and wall thickness of air cells. In Figure 5, the SEM images of the various extrudates are presented to demonstrate their microstructure. The analysis of the images reveals that extruded flour containing higher amounts of SQ displayed consistently larger and more regular pores, measuring 1.7 mm, whereas those made with 75% SGP exhibited more irregular shapes and smaller pores, typically under 1.0 mm. A more uniform porous sponge structure was identified in samples with a SQ: SGP ratio of 75:25%, in contrast to those with a 25:75% ratio, which contained a greater concentration of starch and soluble fibers. The findings align with the studies conducted by Akande et al. (2017) regarding instant grain amaranth‐based extruded flour. Variations in wall thickness corresponded with differences in the uniformity of the cavities. The wall thickness of the extruded flours with higher SQ levels was under 100 μm, whereas those with SGP measured around 170–200 μm (data not presented). These discrepancies in wall thickness were particularly noticeable in the outer layer of the extrudates.

The thickness ranged from 60 to 121 μm for samples with high SQ content and from 263 to 1090 μm for those with higher SGP content. A simple combination of the cross‐sectional and relative longitudinal expansion indicates that the density of extrudates with higher SGP content is higher than that of SQ‐based samples. This agrees well with the peak force results shown in Figure 4 suggesting that materials with a higher SQ content result in softer products. Ciro Baruchs et al. (2024) found that as the amount of starch increases in corn‐based extrudates containing germinated lupin, a greater number of smaller air bubbles with thicker walls contribute to the formation and expansion of air walls. There is a noteworthy correlation between the physical characteristics such as porosity, hardness, and density of samples, as well as the parameters examined in the microscopic analyses (Hashemi et al. 2017). Moreover, increased starch fraction in SQ: SGP 75:25 treatment, combined with higher FMC from 14% to 20%, led to enhanced starch gelatinization, promoting cell size. The outcome aligned with the findings of Cheng et al. (2020).

The optimal formulation for instant flour was selected based on the maximum value of porosity, WAI, AP, and TPC, as well as the minimum hardness and WSI, as important criteria for optimization. Other parameters were kept within the specified range. The desirable conditions were identified as 18.94% FMC and a 75:25 SGP: SQ ratio, yielding a desirability value of 0.898 (Table 4). The predicted values were TPC 14.646 (mg GAE/100 g), AP 34.376 (%), WAI 5.968 g gel/g, porosity 57.763, and hardness 9.345 N. The actual data recorded were TPC 14.01, DPPH 33.56, WAI 5.26, porosity 56.90, and hardness 10.02 N. The t‐test analysis performed using SPSS Statistics revealed that there was no significant difference between the predicted and observed values during validation (*p* ≥ 0.05).

**TABLE 4:** Physicochemical properties of optimal instant flour and untreated flour.

Properties	Untreated flour	Optimal instant flour
Water activity (*a*_*w*_)	0.33^a^ ± 0.68	1.01^b^ ± 0.49
Mean particle size (μm)	0.82^b^ ± 3.38	0.25^a^ ± 4.16
Protein (%)	1.12^a^ ± 16.53	1.19^a^ ± 17.05
Water absorption index (g/g)	0.31^b^ ± 5.88	0.67^a^ ± 6.10
Bulk density (kg/m^3^)	0.740 ± 0.18^a^	0.721 ± 1.55^b^
Total dietary fiber (%)	0.05^b^ ± 3.68	0.13^a^ ± 3.84
Insoluble dietary fiber (%)	1.91^a^ ± 62.95	1.07^a^ ± 61.87
Soluble dietary fiber (%)	0.56^b^ ± 32.35	0.79^a^ ± 34.07
Total phenol content (mg GAE/100 g)	2.26^b^ ± 14.00	1.96^a^ ± 14.69
DPPH (%)	1.47^b^ ± 32.13	1.51^a^ ± 33.81
L*	1.67^a^ ± 86.27	1.20^b^ ± 71.01
a*	0.83^b^ ± 5.91	0.03^a^ ± 6.77
b*	0.61^b^ ± 31.08	0.93^a^ ± 32.84
Dispersibility (%)	0.86^a^ ± 91.11	1.37^b^ ± 92.42
Wetting capacity	NR	2.23 ± 21.76
Carr index	0.39^a^ ± 21.84	0.77^b^ ± 19.97
Hausner ratio	0.57^a^ ± 1.630	0.64^b^ ± 1.428

*Note:* Values are means ± standard deviations; means within a row with different superscript letters (a, b) are significantly different (*p* < 0.05).

Abbreviation: NR, Not Reported.

### 3.8 Quality of the Optimized Extruded Flours Versus the Raw Materials

Table 4 demonstrated that the mean particle size of extruded flour, 4.16 μm was notably greater than that of the untreated one, 3.38 μm. It was found that the BD of the optimized instant flour (0.721 kg/m^3^) was significantly lower than that of the raw materials (740 kg/m^3^). The reduction of BD during extrusion could be caused by structural changes due to the degradation of macromolecules such as carbohydrates and proteins (Ciro Baruchs et al. 2024). Due to starch degradation to short‐chain sugars, the viscosity of the dough was reduced, and consequently, the internal die pressure was lower, leading to an improvement in the cross‐sectional expansion index and a decrease in the bulk density of the final extrudates. This reduced BD of flour could be beneficial for the preparation of low bulk–weaning food (Pismag et al. 2024). A similar result was observed by Albarracín et al. (2019) for whole rice flours modified by germination and extrusion. The WAI (6.10 g/g), SDF (34.07%), TPC (14.69 mg GAE/100 g), and DPPH (33.81%) were higher in the optimally instant flours compared to the untreated samples: 5.88 g/g, 32.35%, 14 mg GAE/100 g, and 32.16% respectively. The extrusion process involves shear forces from screw rotation, which lead to the fragmentation of dietary fiber into lower molecular weight substances, typically resulting in a reduction of the IDF portion while enhancing the soluble fraction (Milani et al. 2024). The increase in WAI can be attributed to a rise in protein levels and changes in protein quality throughout germination and extrusion, as well as the breakdown of polysaccharide structures. This leads to a rise in locations suitable for water interaction and retention (Akande et al. 2017). As a result of the disruption of cell wall matrices and the breakdown of the high molecular weight complex of phenolics during extrusion, the extractability of phenolic compounds is enhanced. A variety of information related to the enhancement of phenolic compounds during extrusion has been released regarding rice flour, soy protein isolate/wheat gluten/corn starch/green tea, and lentil flour/orange peel powder, correspondingly (Albarracín et al. 2019). Moreover, observed that the Rawela cultivar (a deep red type of *Phaseolus vulgaris* ) showed a 14% rise in phenolics in the extrudates compared to the unprocessed beans.

The color characteristics of the extrudates versus raw materials are presented in Table 4. The L* of the extruded flours was 71.01, while the non‐extruded flours exhibited L* = 86.27. The extrusion process also fabricated pre‐cooked flour with increased redness, indicated by higher a* 6.77, as well as greater yellowness, b* 32.84, compared to untreated samples (5.91) and (31.08), respectively. The current finding is in agreement with Ali et al. (2016), who reported a negative impact of extrusion on the lightness of expanded flours. The decrease in L* of extruded flour by increasing baking temperature and screw speed simultaneously was influenced by the breaking of starch bonds and the contribution of more sugars in the browning reactions and hydrolysis pigment degradation (Lotfi Shirazi et al. 2020). Changes in the color of instant flours are generally characterized by non‐enzymatic reactions (Maillard and sugar caramelization) when a reducing sugar interacts with protein amino groups, leading to brown pigmentation. The formation of Maillard browning is highly relevant not only because of its favorable qualities in food production, which yield attractive colors that draw consumer interest. This occurs due to thermal and mechanical impacts, which decompose starches and proteins into simpler elements, causing the reaction to accelerate Maillard reactions (Ciro Baruchs et al. 2024).

The capacity of food particles to become wet without clumping and while allowing agglomerates to disintegrate is a sign of their ability to reconstitute in water, producing a smooth and uniform paste (MMR 2021). Wettability and dispersibility refer to the rehydration processes of instant flours, evaluated as a time function (Barbosa 2021). The wettability of samples indicates how effectively the powdered particles can absorb water on their surfaces, initiating the reconstitution process (Okhravi et al. 2024). The ability of the porridge to disperse in water was assessed to determine its instant reconstitution upon adding water. The parameters of the extrusion process, along with flour composition, particle size, starch gelatinization, and protein denaturation, play a significant role in the formation of agglomerates in water, which are crucial factors influencing wetting capacity and dispersibility (Pismag et al. 2024).

In this investigation, the untreated flours exhibited agglomeration during the wettability test due to their small particle size, making it impossible to assess this parameter. Wettability values for the optimized instant flours were recorded at 21.76. Akande et al. (2017) noted that with an increase in particle size, both powder flowability and dispersibility improved, along with a reduction in the wettability duration, while finer particles tended to clump on the liquid's surface due to strong adhesion forces.

As shown in Table 3, the dispersibility of the optimized instant flour (92.42%) increased slightly compared to the untreated flour (91.13%), which may be attributed to reduced initial moisture content that facilitated improved starch gelatinization and structural breakdown during extrusion. Furthermore, the lower MC of the instant flour contributed to this phenomenon. Recent observations indicate that modified flour has a higher reconstitution under the influence of extrusion and germination. However, these values are lower than previously reported by Comettant‐Rabanal et al. (2021) on extruded whole grain flour and sprout millet.

During the extrusion process, the structures of the granular and crystalline components were altered, resulting in the creation of larger particles with an amorphous form. These modifications greatly improved the wettability and dispersibility of instant flour. As noted by Ali et al. (2016), larger particle sizes and increased porosity enhance the wetting capacity of instant flours, while fine particles are more likely to clump together on the liquid surface due to strong adhesion forces. Nabavi et al. (2024) observed that gluten‐free instant flour made from whole acorn‐maize blends demonstrated better dispersion when processed at lower MC.

To achieve improved flow characteristics of instant powder, the CI% and HR must range from 16% to 20% and 1.19–1.25, respectively (Pismag et al. 2024). Table 4 also indicates the uniform flow properties of the samples, with CI falling from 21.84% for the untreated sample to 19.97% for instant flour. HR measures the cohesive nature of the flour. Compared to untreated flour, HR analyses dropped significantly from 1.630 to 1.428 for extruded flour. The extruded flour showed intermediate cohesiveness, while the untreated sample indicated a low cohesive strength (Akande et al. 2017; Pismag et al. 2024). Overall, in this project, the instant flour showed acceptable flow properties and medium cohesive strength in the CI and HR analyses compared to the optimized formulation of the raw materials. Similar results for CI and HR were reported by Okhravi et al. (2024) on instant powder using whole oat and broken rice flour.

## 4 Conclusion

This study demonstrated the potential of sprouted quinoa (SQ) and sprouted grass pea (SGP) blends to enhance the quality of gluten‐free instant flours through dual modification by sprouting and extrusion. Optimal processing conditions were identified as an 18.94% feed moisture content (FMC) and a 75:25 SGP:SQ ratio, which maximized porosity, water absorption index (WAI), antioxidant activity (AP), and total phenolic content (TPC), while minimizing hardness and water solubility index (WSI). Sprouting enhances nutritional properties, including increased protein, fiber, and antioxidant levels, through the activation of endogenous enzymatic processes. Extrusion further improved functional attributes, including particle size, flowability, and dispersibility, primarily due to starch gelatinization and structural modifications.

The extruded flour exhibited superior physicochemical properties compared to untreated samples, including higher SDF (34.07%), TPC (14.69 mg GAE/100 g), and AP (33.81%), alongside improved color and reconstitution capabilities. Microstructural analysis revealed larger, more uniform pores in SQ‐rich samples, contributing to enhanced texture and lower bulk density. These findings underscore the synergistic effect of sprouting and extrusion in developing nutrient‐dense, gluten‐free products with desirable functional and sensory properties. This research provides a foundation for scaling up the production of innovative gluten‐free flours, particularly for applications in weaning foods and instant porridges.

## Author Contributions

**Mahnoosh Afsharfar:** conceptualization (equal), data curation (equal), formal analysis (equal), funding acquisition (equal), investigation (equal), methodology (equal), project administration (equal), resources (equal), software (equal), validation (equal), visualization (equal), writing – original draft (equal), writing – review and editing (equal). **Zahra BeigMohammadi:** conceptualization (lead), data curation (equal), formal analysis (equal), funding acquisition (equal), investigation (equal), methodology (lead), project administration (lead), resources (equal), supervision (lead), validation (lead), visualization (lead), writing – original draft (lead), writing – review and editing (supporting). **Elnaz Milani:** conceptualization (equal), data curation (equal), formal analysis (equal), funding acquisition (equal), investigation (supporting), methodology (equal), project administration (supporting), resources (equal), supervision (lead), validation (equal), visualization (equal), writing – original draft (supporting), writing – review and editing (supporting). **Seid Mahdi Jafari:** conceptualization (supporting), data curation (equal), formal analysis (equal), funding acquisition (equal), investigation (equal), methodology (supporting), project administration (supporting), resources (equal), software (equal), supervision (supporting), validation (equal), visualization (equal), writing – original draft (supporting), writing – review and editing (supporting).

## Conflicts of Interest

The authors declare no conflicts of interest.

## Data Availability

Additional details regarding the data and conditions for access can be obtained by contacting the corresponding author.
